# Does Immunohistochemistry for Discovered on GIST1 and Minichromosome Maintenance Protein7 Provide Additional Clinicopathological Value in Gastrointestinal Stromal Tumors?

**DOI:** 10.14740/wjon918w

**Published:** 2015-06-12

**Authors:** Dalia Mohamed Abd El-Rehim, Mariana Fathy Gayyed

**Affiliations:** a Department of Pathology, Faculty of Medicine, Minia University, Egypt

**Keywords:** GIST, DOG1, C-KIT, MCM7, Ki-67, Immunohistochemistry

## Abstract

**Background:**

The aim of the study was to investigate the expression of discovered on GIST 1 (DOG1) and minichromosome maintenance protein 7 (MCM7) in addition to the traditional markers, C-KIT and Ki-67, in gastrointestinal stromal tumors (GISTs) to specify the diagnosis and to evaluate their clinicopathological significance in GIST patients.

**Methods:**

Hematoxylin and eosin sections of 43 GISTs were re-examined to review histopathological criteria and risk stratification of these tumors. Immunohistochemistry for DOG1, C-KIT, MCM7, Ki-67 antibodies was performed.

**Results:**

Positive DOG1 and C-KIT expressions were found in 42 (97.7%) and 39 (90.7%) of cases, respectively. DOG1 and C-KIT expression scores were significantly correlated (P < 0.001). Among four C-KIT-negative GISTs, three cases were DOG1-positive. DOG1 was more sensitive and specific than C-KIT in the diagnosis of GISTs. High DOG1 expression scores were significantly associated with tumor size (P = 0.023) and risk (P = 0.037). Significant positive correlation was noted between MCM7 and Ki-67 labeling indices (LIs) (P < 0.001, r = 0.885). MCM7 demonstrated higher proliferation LIs than Ki-67. Significant associations were found between MCM7 and Ki-67 LIs and tumor size (P = 0.001 and 0.003 respectively), mitotic rate (P < 0.001 both) and risk stratification (P < 0.001 both) with a stepwise increase in MCM7 LIs with increasing tumor risk.

**Conclusion:**

DOG1 is an important diagnostic tool for GISTs particularly in C-KIT-negative tumors. It may have a role in GISTs tumorogenesis and progression. Despite the established clinicopathological value of Ki-67 in GISTs, detection of MCM7 expression is recommended as a prognostic adjunct, given its better sensitivity for cellular proliferation and stepwise association with tumor risk.

## Introduction

Gastrointestinal stromal tumors (GISTs) are the most common primary mesenchymal tumors of the gastrointestinal tract [1]. In Egypt, they represent 5.77%, 1.88% and 2.06% of gastric, colon and anorectal malignant tumors respectively [2].

Recently, GISTs have received a lot of attention due to their distinctive biologic behavior, clinicopathological features, underlying molecular mechanisms and treatment modalities. The great majority of GISTs have activating mutations in KIT or the homologous RTK platelet-derived growth factor receptor alpha (PDGFRA) gene. About 9-15% of all GISTs do not exhibit mutations in either KIT or PDGFRA and are now termed “wild type” (WT) for both KIT and PDGFRA [1].

Histologically, GISTs demonstrate considerable morphologic overlap with other tumors. In routine practice, the diagnosis of GISTs is based on the anatomic location of the tumor, histopathology and immunohistochemistry. Immunohistochemistry for C-KIT (CD117) has been used as an ancillary diagnostic measure for GIST diagnosis [3]. However, several studies have reported that 5-10% of GISTs were C-KIT-negative [3, 4]. Moreover, C-KIT was expressed by a number of other tumors that histologically mimic GISTs [5, 6].

The transmembrane protein DOG1 (discovered on GIST 1, anoctamin 1 or TMEM16A.20) has emerged in recent years as a promising biomarker for diagnosis of GISTs, irrespective to the underlying KIT or PDGFRA mutation status or C-KIT expression by immunohistochemistry [4, 7, 8]. The genomic region containing DOG1, 11q13 locus, is amplified in several types of tumors, where it is thought to be involved in tumorogenesis and enhancement of tumor cell proliferation, migration and metastasis [9].

Tumor cell proliferation is highly related to the rate of DNA synthesis and may provide prognostic information. Ki-67 is a reliable proliferative marker that was associated with aggressiveness and recurrence of GISTs [10-12]. However, it does not provide superior prognostic utility over National Institutes of Health (NIH) consensus scheme [10]. Furthermore, assessment of Ki-67 labeling index (LI) is sometimes limited due to suboptimal reproducibility in assessing tumors with a low proliferation capacity [10, 13]. An ideal proliferative marker that exhibits a broader range of expression, providing an objective assessment of proliferative activity, is essentially warranted.

The minichromosome maintenance proteins (MCM), consisting of six members (MCM 2-7), are a family of proteins which form a heterohexameric complex for regulating eukaryotic DNA duplication. MCM 2-7 complex has helicase activity to unwind double stranded DNA and thus helps to initiate DNA replication [14]. MCM proteins are expressed throughout the whole cell cycle, including cells that exit the G0 and enter the G1 phase [15]. Besides their values as cell proliferation markers, the MCM proteins are excellent prognostic and diagnostic markers in various human tumors [16-20].

Of particular interest was the newly addressed oncogenic role of MCM7 in several tumors. It serves as a critical target for oncogenic signaling pathways such as androgen receptor signaling or tumor suppressor pathways such as integrin α7 or retinoblastoma signaling, suggesting it as a potential therapeutic target in tumors [21].

Using immunohistochemistry, the current study aimed at evaluating the sensitivity and specificity of DOG1 as compared with C-KIT in the diagnosis of GISTs. Another goal was to examine MCM7 expression compared to Ki-67 as proliferative indicators in GISTs. The relationships between these markers and different clinicopathological parameters were also evaluated to determine their clinicopathological significance in GIST patients.

## Material and Methods

### Patients’ and tumors’ characteristics

This study included 43 GISTs and 30 non-GISTs diagnosed in Pathology Department, Minia University Hospital and Minia Oncology Center, Egypt during the period from 2005 to 2014. The patients’ mean age was 56.51 ± 9.40 (SD) years and median was 57 years (age range 32 - 75 years). Among cases, 24 (55.8%) were males and 19 (44.2%) were females. The most common complaint was vague abdominal pain. Other uncommon complaints were gastrointestinal bleeding and fatigue. Tumor size ranged from 4 to 31 cm with a median size of 13 cm and a mean of 14.67 ± 6.73 (SD) cm. Data regarding clinicopathological features were summarized in Table 1. Risk stratification of GISTs considering mitotic rate per 50 high power field (HPF), tumor size and anatomic site was done according to the previously published parameters [22].

**Table 1:** Clinicopathological Characteristics of 43 GISTs

Clinicopathological characteristics	n (%)
Gender	
Male	24 (55.8%)
Female	19 (44.2%)
Tumor location	
Gastric	28 (65.1%)
Small intestine	14 (32.6%)
Colon	1 (2.3%)
Tumor type	
Spindle	34 (79%)
Epithelioid	6 (14%
Mixed	3 (7%)
Tumor size	
≤ 5 cm	3 (7%)
> 5 - 10 cm	11 (25.6%)
> 10 cm	29 (67.4%)
Tumor mitotic rate	
≤ 5/50 HPF	24 (55.8%)
> 5/50 HPF	19 (44.2%)
Risk	
Low	9 (20.9%)
Intermediate	11 (25.6%)
High	23 (53.5%)

### Histopathological examination

Hematoxylin and eosin (H&E) sections of GISTs were re-examined in order to review histopathological criteria and risk stratification of these tumors. In addition, 30 tumors in the differential diagnosis of GIST were included as negative controls to test for the specificity of DOG1 and C-KIT. These diagnoses include leiomyosarcoma (three cases), leiomyoma (four cases), malignant peripheral nerve sheath tumor (three cases), schwannoma (three cases), solitary fibrous tumor (two cases), neurofibroma (two cases), melanoma (four cases) and poorly differentiated carcinoma (nine cases). For these non-GISTs, representative H&E-stained slides and previously performed immune-stained slides were reviewed to confirm the diagnoses.

### Immunohistochemistry

Four micrometer thick tissue sections were deparaffinized and rehydrated through xylene and graded ethanol solutions and then blocked for 5 min with 3% hydrogen peroxide to deprive the endogenous peroxidase activity. For antigen retrieval, sections were treated with 0.1 mol/L citrate, pH 6.0, in a 700-W microwave oven for 20 min, and incubated with the primary antibodies for 1 h at room temperature. Primary antibodies include monoclonal C-KIT antibody (clone T595 , ready to use, Leica Biosystems, UK), a monoclonal antibody to DOG1 (clone 1.1, ready to use, ThermoFisher Scientific/Labvision corporation, UK), polyclonal Ki-67 (ready to use, ThermoScientific, UK) and a monoclonal MCM7 antibody (clone DSC-141, diluted at 1:100; Santa Cruz Biotechnology, Santa Cruz, CA, USA). The detection system used was a labeled streptavidin-biotin complex by consecutive application of biotinylated linking antibody and enzyme-conjugated streptavidin for 30 min at room temperature followed by substrate chromogen diaminobenzidine (DAB) for 10 min. Finally, slides were counterstained with hematoxylin, dehydrated through graded ethanol solutions, cleared in xylene and coverslipped. Sections of a previously diagnosed typical case of GIST were used as a positive control for C-KIT and DOG1. Tissue sections of normal tonsils were used as the positive control for MCM7 and Ki-67 antibodies. Negative control was done by omitting the primary antibody.

### Immunohistochemical scoring

Immunoreactivity of both DOG1 and C-KIT was semiquantitatively scored as 0, no staining; 1+, < 5% tumor cells reactive; 2+, 5-25% of tumor cells reactive; 3+, > 25-50% tumor cells reactive; and 4+, > 50% tumor cells reactive. To simplify statistical analysis, tumors with scores 0, 1 and 2 were stratified together as low scores, while those with scores 3 and 4 were considered together as high scores [4, 23].

The MCM7 and Ki-67 LIs were determined by counting number of positive cells in a minimum of 1,000 tumor cells and were expressed as the percentage of positive cells [13].

### Statistical analysis

Chi-square and Fisher’s exact tests were used to compare categorical variables. Mann-Whitney and Kruskal-Wallis tests were conducted to study association of MCM7 and Ki-67 LIs in relation to different clinicopathological variables. Correlation between MCM7 and Ki-67 was evaluated using Spearman’s correlation coefficient. Results were considered statistically significant when P-value ≤ 0.05. Data were analyzed using the Statistical Package for Social Sciences (SPSS) version 16 software.

To determine the diagnostic efficacy of DOG1 and C-KIT in GISTs, the numbers of true-positive (TP), true-negative (TN), false-positive (FP), and false-negative (FN) cases were determined for the markers. Accordingly, the sensitivity, specificity, positive predictive values (PPV), negative predictive values (NPV) and diagnostic accuracy of DOG1 and C-KIT were calculated using MedCalc statistical software.

## Results

### DOG1 and C-KIT expression in GISTs, their diagnostic efficacy and associations with clinicopathological features

**Figure 1:**
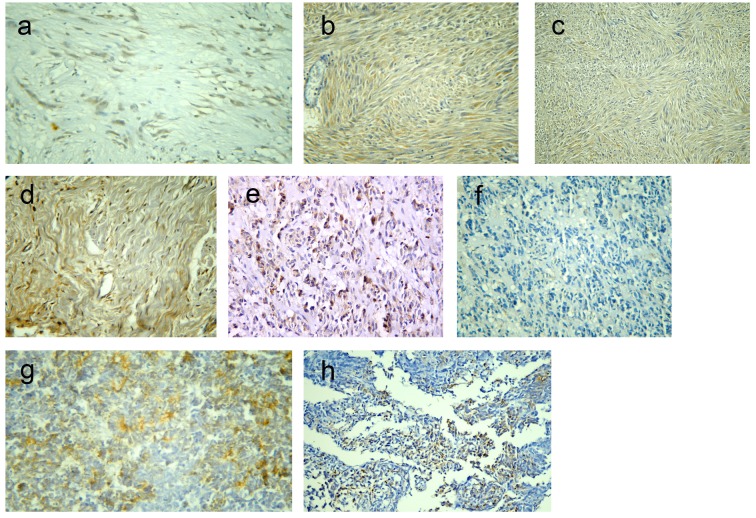
Representative sections of gastrointestinal stromal tumors with positive DOG1 immunostaining: score 2+ (a) and score 4 (b), and positive C-KIT immunostaining: score 2+ (c) and score 4+ (d). A case of epithelioid GIST with positive DOG1 expression and negative C-KIT (e, f). A case of poorly differentiated carcinoma positive for DOG1 expression (g) and a case of leimyosarcoma positive for C-KIT expression (h). Immunohistochemistry, 3,3' diaminobenzidine chromogen and hematoxylin counterstaining. Original magnification × 200 and × 400.

Among 43 GISTs cases, positive DOG1 expression was found in 42 tumors (97.7%) while only one tumor was DOG1-negative. Staining pattern varied from focal to diffuse cytoplasmic expression with or without membranous accentuation (Fig. 1a, b). Sixteen (37.2%) cases had a staining score of 4+, eight (18.6%) cases showed 3+ score, 14 (32.6%) cases showed 2+ score and four (9.3%) cases were 1+. Regarding C-KIT expression, 39 (90.7%) cases were positive, whereas four (9.3%) cases were negative. The staining pattern varied from focal to diffuse cytoplasmic expression with or without membranous accentuation (Fig. 1c, d). Five (11.6%) cases showed score 4+, 10 (23.3%) cases had score 3+, 14 (32.6%) cases were 2+, and 10 (23.3%) cases were 1+. A statistically significant association was found between DOG1 and C-KIT immunostaining scores (P < 0.001) with moderate agreement (kappa = 0.377). However, higher expression scores were more frequently seen in DOG1 than in C-KIT. Among four C-KIT-negative GISTs, three tumors were positive for DOG1 (Fig. 1e, f). DOG1/C-KIT immunoprofiles demonstrated that 39/43 (90.7%) of GISTs showed DOG1+/C-KIT+ immunoprofile, 1/43 (2.3%) had DOG1-/C-KIT- immunoprofile and 3/43 (7%) displayed DOG1+/C-KIT- immunoprofile while no cases had DOG1-/C-KIT+ immunoprofile.

Results regarding the diagnostic efficacy of both markers were presented in Table 2. Both C-KIT and DOG1 had high sensitivity and specificity in diagnosis of GIST; however, DOG1 had higher sensitivity compared to C-KIT. Moreover, DOG1 showed high specificity for GISTs, as only one case of poorly differentiated carcinoma was focally immunoreactive for DOG1 (Fig. 1g), compared to five cases of non-GISTs (one case of leiomyosarcoma (Fig. 1h), two cases of melanoma and two poorly differentiated carcinoma) that showed focal C-KIT-positive expression.

**Table 2:** Diagnostic Efficacy of DOG1 and C-KIT in GISTs

	TP	FN	TN	FP	Sensitivity	Specificity	PPV	NPV	Accuracy
DOG1	42	1	29	1	97.67%	96.67%	97.67%	96.67%	97.26%
KIT	39	4	25	5	90.70%	83.33%	88.64%	86.21%	87.67%

TN: true negative; TP: true positive; FN: false negative; FP: false positive; PPV: positive predictive value; NPV: negative predictive value.

Table 3 demonstrated the association of DOG1 and C-KIT with different clinicopathological variables. Statistically significant associations were found between high DOG1 expression scores and large tumor size (P = 0.023) as well as high risk (P = 0.037) while no significant relation with other variables was identified. No significant difference was found in C-KIT immunostaining scores in relation to clinicopathological features. No significant association was found between both of DOG1 and C-KIT immunostaining scores and the proliferative markers, MCM7 and Ki-67 LIs (Table 4).

**Table 3:** Associations of DOG1 and C-KIT With Clinicopathological Characteristics

Variables	n	DOG1 expression score			C-KIT expression score		
		Score 0, 1, 2	Score 3, 4	P-value	Score 0, 1, 2	Score 3, 4	P-value
Age				0.359			0.219
< 57 years	20	7 (35.0%)	13 (65.0%)		11 (55.0%)	9 (45.0%)	
≥ 57 years	23	12 (52.2%)	11 (47.8%)		17 (73.9%)	6 (26.1%)	
Gender				1.000			0.521
Male	24	11 (45.8%)	13 (54.2%)		17 (70.8%)	7 (29.2%)	
Female	19	8 (42.1%)	11 (57.9%)		11 (57.9%)	8 (42.1%)	
Tumor location				0.453			0.375
Gastric	28	14 (50%)	14 (50%)		19 (67.9%)	9 (32.1%)	
SI	14	5 (35.7%)	9 (64.3%)		9 (64.3%)	5 (35.7%)	
Colon	1	-	1 (100%)		-	1 (100%)	
Tumor type				0.473			0.215
Spindle	34	14 (41.2%)	20 (58.8%)		20 (58.8%)	14 (41.2%)	
Epithelioid	6	4 (66.7%)	2 (33.3%)		5 (83.3%)	1 (16.7%)	
Mixed	3	1 (33.3%)	2 (66.7%)		3 (100%)	-	
Tumor size				0.023			0.121
≤ 5 cm	3	3 (100%)	-		3 (100%)	-	
> 5 - 10 cm	11	7 (63.6%)	4 (36.4%)		9 (81.8%)	2 (18.2%)	
> 10 cm	29	9 (31.0%)	20 (69.0%)		16 (55.2%)	13 (44.8%)	
Tumor mitotic rate				0.217			0.521
≤ 5/50 HPF	24	13 (54.2%)	11 (45.8%)		17 (70.8%)	7 (29.2%)	
> 5/50 HPF	19	6 (31.6%)	13 (68.4%)		11 (57.9%)	8 (42.1%)	
Risk				0.037			0.121
Low	9	6 (66.7%)	3 (33.3%)		8 (88.9%)	1 (11.1%)	
Intermediate	11	7 (63.6%)	4 (36.4%)		8 (72.7%)	3 (27.3%)	
High	23	6 (26.1%)	17 (73.9%)		12 (52.2%)	11 (47.8%)	
Metastasis				1.000			0.692
Yes	8	4 (50%)	4 (50%)		6 (75.0%)	2 (25.0%)	
No	35	15 (42.9%)	20 (57.1%)		22 (62.9%)	13 (37.1%)	

SI: small intestine. Tests of significance: Chi-square test and Fisher’s exact tests. P-value ≤ 0.05 is considered significant.

**Table 4:** Associations of MCM7 and Ki-67 With Clinicopathological Characteristics

Variables	MCM7 LIs			Ki-67 LIs		
	Mean ± SD	Median (min-max)	P-value	Mean ± SD	Median (min-max)	P-value
Age			0.874			0.825
< 57 years	15.90 ± 9.68	12 (3 - 38)		8.05 ± 6.90	6 (0 - 20)	
≥ 57 years	15.52 ± 12.52	13 (0 - 60)		7.82 ± 7.36	6 (0 - 33)	
Gender			0.216			0.101
Male	17.91 ± 13.45	19 (0 - 60)		9.70 ± 8.52	9 (0 - 33)	
Female	12.89 ± 6.67	12 (4 - 30)		5.68 ± 3.78	5 (0 - 13)	
Tumor location			0.926			0.829
Gastric	16.75 ± 13.08	14 (0 - 60)		8.50 ± 7.66	7 (0 - 33)	
SI	13.85 ± 6.31	12 (5 - 26)		7.00 ± 6.11	5 (0 - 19)	
Colon	12	12		5	5	
Tumor type			0.288			0.099
Spindle	15.08 ± 12.13	12 (0 - 60)		7.08 ± 7.25	5 (0 - 33)	
Epithelioid	16.83 ± 7.16	17 (7 - 25)		9.50 ± 5.82	8 (4 - 19)	
Mixed	15.69 ± 11.16	18(18 - 25)		14.33 ± 4.04	15 (10 - 18)	
Tumor size			0.001			0.003
≤ 5 cm	10.66 ± 2.30	12 (8 - 12)		2.66 ± 2.30	4 (0 - 4)	
> 5 - 10 cm	7.27 ± 6.52	6 (0 - 23)		3.54 ± 4.52	4 (0 - 15)	
>10 cm	19.41 ± 11.24	18 (5 - 60)		10.13 ± 7.19	10 (0 - 33)	
Tumor mitotic rate			< 0.001			< 0.001
≤ 5/50 HPF	9.66 ± 5.56	10 (0 - 20)		3.62 ± 3.29	4 (0 - 10)	
> 5/50 HPF	23.31 ± 11.88	23 (9 - 60)		13.36 ± 6.84	13 (4 - 33)	
Risk			< 0.001			< 0.001
Low	5.00 ± 3.84	5 (0 - 12)		2.00 ± 2.44	0 (0 - 6)	
Intermediate	12.63 ± 4.41	12 (5 - 20)		4.36 ± 3.32	5 (0 - 10)	
High	21.34 ± 11.78	20 (5 - 60)		11.95 ± 7.11	12 (0 - 33)	
Metastasis			0.132			0.381
Yes	18.75 ± 8.37	19 (5 - 30)		9.12 ± 5.71	10 (0 - 19)	
No	15.00 ± 11.69	12 (0 - 60)		7.65 ± 7.39	5 (0 - 33)	
DOG1 score			0.339			0.123
Score 0, 1, 2	15.36 ± 13.56	12 (0 - 60)		6.89 ± 8.40	4 (0 - 33)	
Score 3, 4	15.95 ± 9.12	13 (0 - 38)		8.75 ± 5.86	7 (0 - 20)	
C-KIT score			0.548			0.572
Score 0, 1, 2	15.60 ± 12.60	12 (0 - 60)		7.75 ± 7.78	5 (0 - 33)	
Score 3, 4	15.86 ± 8.19	13 (5 - 33)		8.26 ± 5.75	6 (0 - 20)	

SI: small intestine. LIs: labeling indices (%). Tests of significance: Mann-Whitney and Kruskal-Wallis tests. P-value ≤ 0.05 is considered significant.

### MCM7 and Ki-67 expression in GISTs and their associations with clinicopathological features

**Figure 2:**
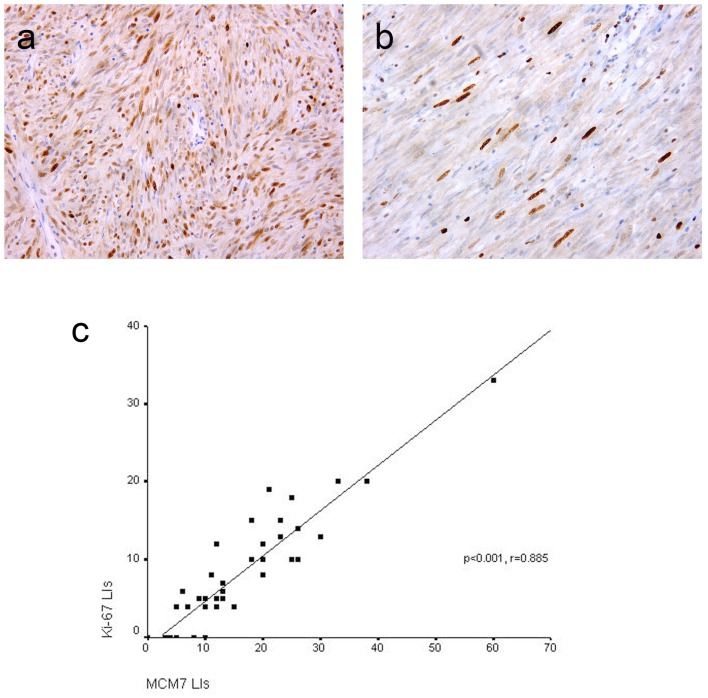
Representative sections of gastrointestinal stromal tumors with positive MCM7 (a) and Ki-67 (b) expression with high and low LIs respectively. Immunohistochemistry, 3,3' diaminobenzidine chromogen and hematoxylin counterstaining. Original magnification × 400. Significant positive correlation between MCM7 and Ki-67 labeling indices (LIs) in gastrointestinal stromal tumors (c).

MCM7 and Ki-67 immunoreactivity was noted in the nuclei of the tumor cells (Fig. 2a, b). Mean ± SD LIs of MCM7 and Ki-67 were15.69 ± 11.16 and 7.93 ± 7.07, respectively and median LIs were 12 (range 0 - 60) and 6 (range 0 - 33), respectively. As shown in Figure 2c, significant positive correlation was found between MCM7 and Ki-67 LIs (P < 0.001, r = 0.885). However, MCM7 demonstrated higher tumor LIs than Ki-67.

Significant associations were noted between MCM7 and Ki-67 LIs and tumor size (P = 0.001 and 0.003 respectively), mitotic rate (P < 0.001 both) and risk stratification (P < 0.001 both). No association associations were observed in relation to other clinicopathological parameters (Table 4).

## Discussion

DOG1 and C-KIT have been reported to be the markers of choice in the diagnosis GISTs, and were highly expressed in most GISTs in either focal or diffuse patterns [23-25].

Comparable to published data [8, 26], DOG1/C-KIT immunoprofiles in this series demonstrated that 90.7% of GISTs showed DOG1+/C-KIT+ immunoprofile, 2.3% had DOG1-/C-KIT- immunoprofile and 7% displayed DOG1+/C-KIT- immunoprofile, whereas no cases with DOG1-/C-KIT+ immunoprofile were detected. However, a previous study reported DOG1-/C-KIT+ immunoprofile in 3% of GISTs [26].

In this series, the expressions of DOG1 and C-KIT were generally concordant. However, the expression pattern of DOG1 was more diffuse compared to C-KIT. In many cases focal C-KIT positivity was noticed while DOG1 staining was more diffuse and thus easier to interpret and reliable than C-KIT. Among four C-KIT-negative GISTs, three cases were DOG1-positive. This was in agreement with previous studies that identified a considerable proportion of DOG1-positive GISTs in C-KIT-negative cases [4, 24, 27]. These findings together with ours suggest that the use of DOG1 in C-KIT-negative cases will be very helpful in the diagnosis of such cases.

On comparing the diagnostic efficacy of DOG1 with that of C-KIT in GISTs, the current study showed that DOG1 and C-KIT were sensitive and specific markers for GISTs; however, DOG1 had superior sensitivity and specificity for GIST diagnosis than C-KIT. Others reported a nearly equal sensitivity of DOG1 and C-KIT with higher DOG1 specificity in diagnosis of GISTs [23, 28]. In agreement with previous studies [4, 7, 24], the present study detected focal positive DOG1 expression in only one case of non-GISTs while C-KIT immunoreactivity was observed in 5/30 cases of non-GISTs. Our finding was also supported by Lee and his coauthors who reported that DOG1 immunoreactivity was rarely observed in other mesenchymal and non-mesenchymal tumors [26] with a negative to a very low false-positive rate less than 1% in non-GISTs [7, 23, 24, 29], suggesting that DOG1 is a highly specific and sensitive immunohistochemical marker of GISTs.

Consistent with previous literature (reviewed in Lee et al 2010) [26], the majority of GIST cases studied in the present study were positive for both C-KIT and DOG1 and none of the non-GISTs examined in this study had shown immunohistochemical co-expression of both markers. Therefore, concurrent C-KIT, and DOG1 immunoreactivity is efficiently diagnostic of GIST.

DOG1 overexpression and amplification of the chromosomal band 11q13, the genomic region containing DOG1, is frequently seen in several tumors and was associated with aggressive features and poor prognosis in some of these tumors. These findings were recently confirmed by experimental studies that showed association between DOG1 amplification and increased proliferation, invasion and metastasis of tumor cells [9, 30]. Likewise, C-KIT expression was documented in multiple solid neoplasms, where its expression was correlated with aggressive tumors and poor prognosis [31-33].

Little and somewhat conflicting information was available in literature regarding the association between GIST-associated proteins (C-KIT and DOG1) and clinicopathological characteristics in GISTs. In the present study, high DOG1 expression score was significantly associated with high risk and large size tumors. This correlation was not established with C-KIT. In agreement with these findings, a previous study found that DOG1 expression, but not C-KIT, was significantly associated with risk of tumors [25]. Another study found that the high C-KIT and DOG1 expression scores were significantly associated with high-risk tumors and only C-KIT expression was significantly associated with large tumor size [23]. On the contrary, lack of significant association between C-KIT or DOG1 expression and the risk of malignancy was found while significantly correlated with tumor spindle cell morphology [34]. Consistent with our findings, several studies reported lack of correlation between both markers and tumor location [24, 34, 35]. However, a statistically significant association between high DOG1 scores and gastric GIST was demonstrated [23]. Others reported a negative correlation between DOG1 expression and mitotic count [8], tumor recurrence and/or metastasis [27]. These reports seem to contradict each other; however, the restricted number of cases, the different antibodies, scoring systems and cutoff used by various studies may be responsible for these discrepancies.

A link between DOG1 expression and cell-cycle regulation and proliferation has recently emerged in several tumors. Its effect on the proliferation of interstitial cells of Cajal (ICC), the putative cell of origin of GIST has recently been reported [9, 36]. This raises the question of whether DOG1 has a similar role in regulating proliferation of GISTs. The current study demonstrated lack of significant associations between DOG1 and C-KIT expression and the proliferative markers, MCM7 and Ki-67. This goes in line with a recent *in vitro* study which demonstrated that DOG1 had small effects on cell proliferation in GISTs while its inhibition had a pro-apoptotic role on some early apoptotic GIST cell populations [37]. Further larger studies are warranted to fully elucidate the role of DOG1 on cellular proliferation and its potential as therapeutic target in GIST patients.

Previous studies have reported Ki-67 and MCM proteins as good prognostic and diagnostic markers in different human tumors. Several studies have proved a greater reliability of MCM proteins to stain proliferating cells compared to Ki-67 and demonstrated higher sensitivity and specificity of MCM proteins than Ki-67 in various tumors [15-20].

One of the main aims of this study was to compare MCM7 and Ki-67 reproducibility in assessment of proliferative activity and to evaluate their clinicopathological values in GISTs. Despite the highly significant linear correlation found in this study between MCM7 and Ki-67 LIs, a considerably higher proportion of proliferative cells were detected using MCM7 immunohistochemistry compared to Ki-67. Assessment of Ki-67 LI was somewhat limited by its suboptimal sensitivity in some cases, as shown here, by sparse immunoreactivity in low and intermediate risk GISTs. This is probably reflecting cells in the early G1 phase that failed to be labeled by Ki-67 while stained positive for MCM7. MCM proteins expression is seen during all phases of cell cycle, including early G1 phase, and may thus better represent the rate of cell proliferation [15].

Ki-67 and MCM7 LIs were both significantly associated with increasing tumor size, mitotic rate and risk. In addition, a stepwise increase in MCM7 LIs in relation to tumor risk was more frequently seen than in Ki-67. These findings are consistent with previous studies that reported significant associations between one of the MCM family members, MCM2 LIs and high tumor risk [10] and between increased Ki-67 LIs and tumor mitotic activity [12, 38], size [12], risk [10, 12] and relapse [11].

Accordingly, this study suggests that MCM7, albeit does not provide superior clinicopathological value over Ki-67, it can still be considered as a helpful prognostic marker for GISTs, given its higher sensitivity for proliferating cells than Ki-67 and a more stepwise association with increasing risk level. Therefore, simultaneous detection of MCM7 expression in GISTs may provide a more objective assessment and better prediction of clinical aggressiveness.

### Conclusion

Our findings suggest that DOG1 should be added into the diagnostic panel evaluating GISTs and other histologically mimics tumors. The significant association, shown in the current study, between DOG1 expression with tumor size and risk together with its reported correlation with some of the risk group indicators in literature suggests that DOG1 has not only diagnostic but prognostic utility as well. However, further studies with a larger scale of tumors are warranted to characterize the usefulness of DOG1 as a prognostic marker. Evaluation of MCM7 expression in GISTs may provide a more objective assessment of cellular proliferation and better prediction of tumor aggressiveness.
